# Screening for Cognitive Function in Complete Immobility Using Brain–Machine Interfaces: A Proof of Principle Study

**DOI:** 10.3389/fnins.2018.00517

**Published:** 2018-08-15

**Authors:** Dorothée Lulé, Katharina Hörner, Cynthia Vazquez, Helena Aho-Özhan, Jürgen Keller, Martin Gorges, Ingo Uttner, Albert C. Ludolph

**Affiliations:** Department of Neurology, University of Ulm, Ulm, Germany

**Keywords:** brain–machine interface, brain–computer interface, cognition, ECAS, neuropsychology, P300, oddball, amyotrophic lateral sclerosis

## Abstract

**Background:** In many neurological conditions, there is a combination of decline in physical function and cognitive abilities. For far advanced stages of physical disability where speaking and hand motor abilities are severely impaired, there is a lack of standardized approach to screen for cognitive profile.

**Methods:** *N* = 40 healthy subjects were included in the study. For proof of principle, *N* = 6 ALS patients were additionally measured. For cognitive screening, we used the Edinburgh cognitive and behavioral ALS screen (ECAS) in the standard paper-and-pencil version. Additionally, we adapted the ECAS to a brain–machine interface (BMI) control module to screen for cognition in severely advanced patients.

**Results:** There was a high congruency between BMI version and the paper-and-pencil version of the ECAS. Sensitivity and specificity of the ECAS-BMI were mostly high whereas stress and weariness for the patient were low.

**Discussion/Conclusion:** We hereby present evidence that adaptation of a standardized neuropsychological test for BMI control is feasible. BMI driven neuropsychological test provides congruent results compared to standardized tests with a good specificity and sensitivity but low patient load.

## Introduction

In many neurological conditions such as amyotrophic lateral sclerosis (ALS), there is a combination of decline in physical function and cognitive abilities. About 50% of ALS patients present with cognitive deficits which are mostly mild and restricted to one cognitive domain; only 5–15% present with full blown fronto-temporal dementia (FTD; Phukan et al., 2012; Goldstein and Abrahams, 2013). In several studies on ALS, there has been evidence for specific impairments in fluency, language, executive function including social cognition, and verbal memory (Beeldman et al., 2016). It has been discussed controversially whether these impairments decline in the course of physical function loss. Whereas some find no evidence (Kasper et al., 2016; Xu et al., 2017), others report cognitive decline in the course of the disease (Elamin et al., 2013; Trojsi et al., 2016), but possibly only in a subsets of patients, e.g., with bulbar onset (Schreiber et al., 2005). Discrepancies between studies could either be explained by different subgroups or by a training effect in a retest design. Most importantly, most studies so far did not use motor adapted neuropsychological tests which can be performed either written or verbally. The Edinburgh cognitive and behavioural ALS screen (ECAS) as a standardized test with parallel versions to be performed either written or verbally was a first approach to bridge this knowledge gap (Abrahams et al., 2014). Thus, for mildly advanced stages of physical impairments, standard neuropsychological tests are at hand. For moderately advanced stages of motor decline, eye-tracking controlled devices can be used for neuropsychological screening (Keller et al., 2015). However, for far advanced stages of physical disability where speaking and hand motor abilities are severely impaired, a state referred to as locked-in state, standardized approaches to measure cognitive function are lacking. Instead, in this state, there have been single case studies on cognitive profiles only, using near infrared spectroscopy (Fuchino et al., 2008), event-related potentials (ERPs; Kotchoubey et al., 2003; Ogawa et al., 2009) or visual recording of eye-blink responses (Lakerveld et al., 2008). Cohort studies have not been performed so far and there is lack of informative data on the cognitive abilities of the vast majority of these patients. Instead, many locked-in (LIS) patients including those with ALS are clinically regarded to have dementia despite no valid data on cognitive profile. Single case studies provide evidence for preserved cognitive function in LIS, e.g., Lakerveld et al. (2008) tested for memory and attentional abilities in LIS by asking the patient to respond via eye blink which was visually detected by the interviewer; they provided evidence for superior cognitive abilities in LIS. However, only recently have there been standardized approaches to screen for cognitive abilities in far advanced stages of motor impairments. First paradigms used state-of-the-art eye-tracking controlled setups in lab environment (Cipresso et al., 2012; Keller et al., 2015) and at bedside (Keller et al., 2017) to reliably detect cognitive impairment. LIS state Brain–machine interfaces (BMIs) have been widely used for patients with severe physical restriction for environment control (Mugler et al., 2010; Münßinger et al., 2010) and communication (Nijboer et al., 2008) and might provide additional information on state of alertness in complete LIS state (Chaudhary et al., 2016). BMIs might also be used to screen for cognitive function which has been tested in ALS for single cognitive domains already (Poletti et al., 2016). We hereby present a unique approach for the use of BMIs to conduct a standardized neuropsychological screening method on several cognitive functions in patients with ALS. We hereby use a commercially available EEG device which has been shown to be sufficient for BMI communication (Duvinage et al., 2013) and combine it with the widely used ECAS to enable clinicians and researchers to screen for disease specific cognitive functions to bridge the gap of knowledge with regard to cognitive profile in complete immobility.

## Materials and Methods

### Subjects

In total, *N* = 40 healthy controls were included who were matched to ALS patients with regard to age, gender, and education according to previous studies (Keller et al., 2015). To test for feasibility in physically impaired patients, *N* = 6 ALS patients were included (**Table 1**). None of the participants had signs of any neurological or psychiatric illness (other than ALS) or dementia. They were all native German speakers. Patients were consecutively recruited from the clinics of the Department of Neurology at the Universitätsklinikum Ulm, Germany. The study was approved by the Ethics Committee of the University of Ulm (No. 19/12). All participants gave written informed consent to the study according to institutional guidelines.

**Table 1:** m, male; f, female; ADI-12, ALS depression inventory 12 items; ALS-FRS-R, ALS-Functional Rating Scale – revised form ranging from 0 to 48, where 0 indicates complete immobility.

	Controls (*N* = 40)			ALS patients (*N* = 6)			*p*
			
	Mean	*SD*	Range	Mean	*SD*	Range	
Age	61.2	6.9	44–72	56.2	4.3	53–64	0.06
Gender (m/f)	15/25			4/2			0.36
Education years	15.7	2.7	8–20	12.8	1.3	12–15	0.01^∗^
ADI-12	16.75	3.7	12–26	22.3	9.3	16.41	0.03^∗^
Site of onset (Spinal/bulbar)				5/1		
Months since onset				13.7	8.9	6–27
ALS-FRS-R				40^§^	4.5	33–46


*^§^indicates moderate physical impairment. Chi^2^ test for gender and site of onset and Mann–Whitney U-test for all other comparisons were used. ^∗^*p* < 0.05.*

### Study Design

First, participants were screened for affective (ALS depression inventory 12 items, ADI-12; Kübler et al., 2005; Hammer et al., 2008), physical (ALS functional rating scale revised version, ALS-FRS-R; Cedarbaum et al., 1999), and cognitive function [German version of the Edinburgh cognitive and behavioral ALS screen (ECAS); Abrahams et al., 2014; Lulé et al., 2015; Loose et al., 2016] by a board certified neuropsychologist. In randomized order, half of the participants performed the ECAS in a standard paper-and-pencil version first (ECAS parallel version C) and then the adapted BMI ECAS version (ECAS original version A), whereas the other half performed both versions in reverse order. The procedure took about 2 h.

### BMI Setup

For bedside BMI neuropsychological testing, the mobile BMI device Neuroheadset Emotiv Epoc+ was used (AF3, F7, F3, FC5, T7, P7, O1, O2, P8, T8, FC6, F4, F8, AF4, reference, for further information see www.emotiv.com). Electrode impedance was decreased by using saline liquid until the level required by the software was reached (in the 10–20 kOhm range) and was checked along the experiment.

**FIGURE 1:**
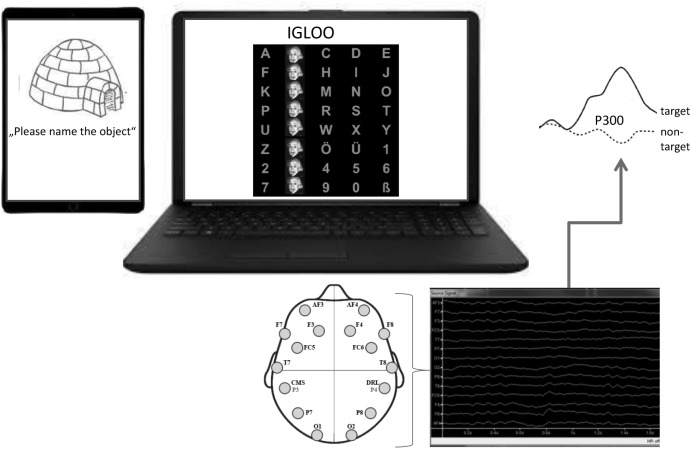
BMI set-up. **Left:** Positions of the 14 electrodes of the Emotiv Epoc headset according to the 10–20 system. **Middle:** 8 × 5 speller matrix for the P300 speller. Rows and columns of symbols were disguised for 62.5 ms by faces (Albert Einstein) with a 200 ms interstimulus interval signal.

Participants were positioned in front of a 8 × 5 speller matrix adapted according to Farwell and Donchin (1988) which was presented on a laptop screen (letters A–Z, German “Umlaute”, ß, digits 0–9). Rows and columns of symbols were disguised for 62.5 ms by faces (a face of Albert Einstein) with a 125 ms interstimulus interval (Kaufmann et al., 2013; **Figure 1**). Participants were asked to fixate a target which was then highlighted twice (rare event eliciting a P300) in a row of 11 non-target highlight events. Selected stimulus according to P300 was presented above the speller matrix.

Each session was composed of one calibration set and one ECAS trial. Calibration of the Speller was performed by asking the participant to spell the sentence “Ulm is nice” (“Ulm ist schön”) with the BMI. During this run, participants received no feedback (i.e., subjects did not see which character the system actually selected), since data were only collected for system calibration. The percentage of letters correctly selected by the system out of the phrase, considered as the measure of BMI calibration (‘BMI calibration accuracy’) was automatically calculated. Only when BMI calibration accuracy’ was >85%, ECAS BMI was performed. In this study, all participants were above this accuracy threshold.

### BMI Data Analysis

EEG data were recorded with the freeware BCI2000 (Schalk et al., 2004). Using an oddball paradigm, a P300 signal was measured. BCI200 classifier was used to determine P300 signal as a positive deflection in voltage (up to 5 μV) with a latency of 800 ms from the stimulus onset. The sampling rate was 128 Hz. The EEG signal was high-pass filtered at 1 Hz and analysed offline with a common average reference (CAR) spatial filter.

### Correctness of the ECAS BMI Selection

Participants were asked to verbally indicate to the investigator when the selected item by the system was not the intended one. The percentage of correctly selected items by the system was recorded.

### Stress and Weariness Rating

Following the BMI ECAS version, participants were asked to rate their emotional stress and weariness following the ECAS BMI use on a 5 point Likert scale.

### Paper-and-Pencil Version of the ECAS

The ECAS is a widely used and well-validated ALS specific cognitive screening tool measuring five domains of ALS specific (executive function, language, and verbal fluency) and non-ALS specific cognitive functions (memory and visuospatial perception; Abrahams et al., 2014; Lulé et al., 2015; Loose et al., 2016^1^). In total, the ECAS encompasses 15 subtasks which are subsumed under the five domains. Maximum total ECAS score is 136 with decreasing score indicating lower cognitive performance.

### BMI Version of the ECAS

For the BMI adaptation, specific subtasks of the original ECAS were selected such as language (naming and language comprehension), restricted phonematic fluency and executive functions (sentence completion and social cognition) for the ALS specific tasks. For the non-ALS specific tasks, memory (immediate recall and delayed recognition, key words of the ECAS instead of whole story) and visuospatial function (cube counting) was selected. For the patient BMI-ECAS version, the length of the test needed to be reduced by selecting the most discriminative items in the text according to previous research (Lulé et al., 2015). According to performance in healthy subjects, tables for verbal fluency scores and cut-off scores for cognitive impairments were defined according to ECAS criteria (<2 SD from mean for cognitive impairments; Abrahams et al., 2014; **Table 2**).

**Table 2:** Congruency of Standard paper and pencil ECAS version and BMI adapted ECAS version.

	BMI naming	BMI comprehension	BMI fluency	BMI sentence completion	BMI social cognition	BMI immediate recall	BMI delayed recall	BMI cube counting
**Standard naming**	-0.076	–	0.081	-0.114	0.256	-0.135	-0.058	-0.065
**Standard comprehension**	–	–	–	–	–	–	–	–
**Standard fluency**	0.042	–	0.321^*^	0.248	-0.051	0.178	0.040	-0.010
**Standard sentence completion**	-0.069	–	0.206	0.329^*^	0.036	0.301	0.092	-0.105
**Standard social cognition**	0.364^*^	–	0.061	0.232	0.678^**^	0.382^*^	0.066	0.465^**^
**Standard immediate recall**	-0.103	–	0.144	0.028	0.299	0.300	-0.022	0.353^*^
**Standard delayed recall**	-0.018	–	-0.208	0.212	-0.088	0.152	0.160	0.076
**Standard cube counting**	0.060	–	0.032	0.300	0.344^*^	0.447^*^	0.171	0.355^*^


*Language comprehension is not possible to correlate as variance in values is too low; Spearman-Rho correlation with ^∗^*p* < 0.05, ^∗∗^*p* < 0.01.*

Naming: scorpion and igloo had to be named (maximum 2 points; original ECAS 8 objects).

Language comprehension: 4 objects were presented numbered 1–4. Four sentences (original ECAS, 8 sentences) were acoustically presented of which one sentence described a property of one object each. Participants were asked to select the correct object for each consecutively presented sentence (maximum 4 points; original ECAS, 8 objects and 8 sentences).

Memory: 10 words (all words to be remembered from the original ECAS) were acoustically presented. Following, participants were asked to produce all words which they could remember (immediate recall). For delayed recognition, ten words were presented of which only 5 had actually been presented before. Subjects had to indicate “y” for yes or “n” for no according to whether this word had been presented before (maximum 10 points; original ECAS, a story is given but only the ten words of the BMI test are valid for scoring in the original ECAS).

Visuoconstruction: Two objects that were made of cubes were presented separately. Subjects had to determine the number of cubes of each object (maximum 2 points; original ECAS, 4 objects).

Sentence completion: three sentences with the last word missing were to be completed by the subject by providing a word which did not logically complete the sentence (maximum 3 points; original ECAS, 6 sentences).

Restricted phonematic fluency: subjects had to name 4 letter words with the given initial letter “G” within 8 min (maximum 12 points; same for original ECAS within 90 s time). Verbal fluency index was calculated according to healthy subjects’ performance analogous to Abrahams et al. (2014).

Social cognition: subjects first had to indicate personal preference for one out of four numbered objects. Three sets of four objects were presented (original ECAS six sets of four objects). Subsequently, subjects had to indicate the preference of a face that was presented adjacent to the same sets of four objects (maximum 6 points).

All answers were spelled via the spelling matrix of the P300 speller (**Figure 1**).

According to performance in healthy subjects, tables for verbal fluency scores and cut-off scores for cognitive impairments were defined according to ECAS criteria (<2 SD from mean for cognitive impairments; Abrahams et al., 2014; **Table 2**).

### Statistics

Data were managed in SPSS (SPSS version 21.0 IBM). Mann–Whitney *U*-test was used for group comparison with effect size *r*. For correlation analysis, Spearman–Rho test was used. All analyses were two-sided and significance level was set at *p* = 0.05.

## Results

### General Cognitive Screening

When compared to healthy controls, patients scored significantly lower in the language function (*U* = 65.00, *z* = -2.22, *p* = 0.027, *r* = -0.33). Scores of the other domains (executive function, verbal fluency, memory, and visuospatial function) did not significantly differ between the groups (all *p* > 0.05).

### Congruence of BMI and Paper–Pencil ECAS

To determine whether performance accuracy of the BMI ECAS could associate performance accuracy of the written paper–pencil version, a Spearman–Rho correlation analysis was performed, showing a significant correlation between the performance in both versions of healthy controls (Spearman–Rho *r* = 0.64, *p* < 0.001) and of both groups (Spearman–Rho *r* = 0.51, *p* < 0.001). For patients, congruency was also acceptable but did not reach significance due to the small sample size (Spearman–Rho *r* = 0.40, *p* = 0.43).

**FIGURE 2:**
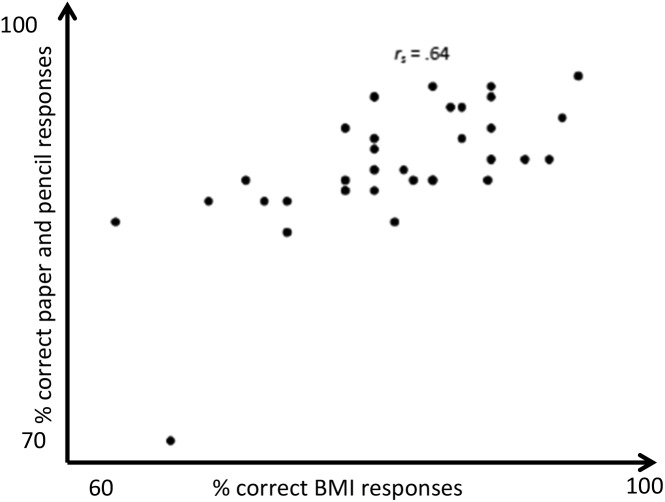
Congruency of ECAS paper and pencil version and BMI version in healthy controls.

For different cognitive functions, there was a significant congruency for verbal fluency, sentence completion, social cognition, immediate recall, social cognition, and cube counting, for the other cognitive functions, congruency was low (**Figure 2** and **Table 3**).

**Table 3:** Predictive validity of the ECAS BMI version.

	Max		Cut-off						Specificity [%]	Sensitivity [%]	PPV [%]
									
	BMI	Paper pencil	BMI	Paper pencil	a	b	c	d			
Language	6	28	5.6	25.8	0	4	1	35	90	0	0
Fluency	12	24	8.5	17.9	2	5	0	33	87	100	29
Executive	12	48	10.6	39.2	2	4	4	30	88	33	33
ALS specific	30	100	23.6	84.3	2	3	0	35	92	100	40
Memory	14	24	6.7	18.8	3	3	2	32	91	60	50
Visuospatial	2	12	1.7	10.9	1	2	3	34	94	25	33
Non-ALS specific	16	36	8.7	30.1	4	2	5	29	94	44	67
ECAS total	46	136	32.9	115.8	1	2	1	36	95	50	33


*Cut-offs were defined according to performance in healthy subjects (<2 SD of mean score). BMI, brain–machine interface ECAS version; Paper Pencil, paper and pencil ECAS standard version; for a–d, cognitive impairment was defined as <cut-off for both BMI and paper and pencil version with a, correct positives; b, false positives; c, false negatives; d, correct negatives; PPV, positive predictive value.*

### Validation of ECAS BMI Version in Healthy Controls

Cognitive impairments for the total score and the five domains were determined according to cut-off scores. Overall, one healthy subject performed 2 SD below the overall ECAS score and for the domain language, fluency, and executive function. Memory and visuospatial performance was impaired in three and four subjects, respectively.

### Sensitivity and Specificity of the ECAS BMI Version

There was a high convergent validity of the BMI version of the ECAS, especially for fluency, sentence completion, social condition, and cube counting. Predictive validity of the ECAS BMI version was good. This was seen in a high sensitivity, specificity, and positive predictive values, especially for social cognition and verbal fluency (**Table 2**). The functions immediate recall and cube counting in addition to the domain executive function showed high specificity whereas sensitivity was in a medium range. Only language showed low sensitivity and positive predictive value. The overall specificity of the BMI version compared to the paper-and-pencil version was very high at 95%.

### Correctness of the ECAS BMI Selection

The median of correct answers of the control group for the P 300 speller was 85% for healthy subjects and 86% for ALS patients.

### Subjective Rating of the Test

Patients (88%) and healthy subjects (86%) mostly reported that they were not stressed by the ECAS BMI version and only a minority reported to be slightly stressed by the procedure (12 and 14%, respectively); 48% of healthy subjects and 18% of ALS patients regarded the BMI procedure to be wearisome.

## Discussion

So far, little is known about the cognitive state in complete immobility in the course of physical decline in ALS (Fuchino et al., 2008; Lakerveld et al., 2008). BMIs have been mainly used to communicate with the patients to unlock the patients mind. We hereby present a new BMI approach for neuropsychological assessment in physically severely handicapped patients. Using this approach, there is a standardized way to measure the cognitive profile in these subjects.

We used a mobile P 300-based BMI algorithm to drive an ALS specific neuropsychological test, the ECAS. Patients presented a reduced performance in the language function in the paper-and-pencil version compared to healthy controls. This is in line with the previous findings that language function is the most sensitive cognitive ability in the course of ALS (Keller et al., 2015; Lulé et al., 2015; Niven et al., 2015; Wei et al., 2015). For executive and visuospatial functions there was a trend but other cognitive functions were not significantly different between groups which was mainly attributed to small sample size. There was a high congruency (Schmidt-Atzert and Amelang, 2012) between the adapted ECAS BMI and the original version. Lack of congruency for some functions might be explained by the adaptations in the BMI versions, mainly the reduction of items. The ECAS BMI version showed a high convergent and predictive validity. This was indicated by a high sensitivity, specificity, and positive predictive value, especially for social condition and verbal fluency. Functions of the domains memory and visual spatial abilities, and executive functions showed high specificity whereas sensitivity was in a medium range. Only language showed low sensitivity and low positive predictive value which was partly explained by the fact of low numbers of impaired controls in this domain. Interestingly, specificity of single functions was similarly high as for the domain itself. This implies that the measurement of one single function was sufficient to get an overall estimation of the cognitive domain. Future studies in larger samples are needed to verify this hypothesis.

The correctness of the P300 speller was 85% in healthy subjects and 86% in ALS patients, fulfilling the criterion of a minimum of 70% accuracy as a predictor for satisfactory communication (Choularton and Dale, 2004) and above the level of far advanced ALS patients in other studies (Marchetti and Priftis, 2014). Accordingly, patients were just as precise in spelling as the healthy subjects (McCane et al., 2015), despite that a commercial EEG device was used. For scientific P300 EEG analysis, there are more open source products available which might better suit these purposes than the hereby used Emotiv Epoc. However, for satisfying communication with BMI, an accuracy rate of 70% is required which was achieved by the hereby presented approach. Due to a high intrinsic motivation to learn BMI control for the future, the patients might have been especially concentrated during the task. Overall, the ECAS BMI seems to be a feasible way to easily and reliably detect cognitive deficits in ALS, especially since most subjects rated the BMI version to be valid and neither stressful nor explicitly strenuous.

The major limitation of the current study is the lack of validation in a large patient sample with severe physical impairments. We hereby present a first proof of principle design with promising results but future studies in patients with advanced physical impairments are warranted. Another limitation is that most tasks of the original ECAS were simplified and shortened and therefore not identical to the original version. Due to high congruency of both of versions, it can be assumed that both approaches measure similar cognitive constructs. However, in future trials similar ECAS versions need to be used for BMI and paper and pencil versions.

## Conclusion

In this proof of principal study, we provide evidence that neuropsychological screening can be performed using BMI algorithms, even with off-the-shelf commercially available EEG systems. So far, the studies are incongruent whether there is cognitive decline in the course of physical function loss (Schreiber et al., 2005; Elamin et al., 2013; Trojsi et al., 2016) or not (Kasper et al., 2016; Xu et al., 2017). In which way cognitive function develop, especially in the final state of physical function decline, is so far mostly unknown. The main target of future trials will be to see whether BMI controlled cognitive screening methods are superior to previously introduced methods with eyetracking control for those patients with residual eye movement (Keller et al., 2016). For patients in complete locked-in state, BMI driven approaches are a cost-effective and simple means of neuropsychological examination of CLIS patients (Poletti et al., 2016). The hereby presented BMI version of a standard neuropsychological test is the next milestone to learn more about cognitive decline in the course of ALS (Cipresso et al., 2012; Poletti et al., 2016) but future studies are required to further develop this approach.

## Author Contributions

All the authors have participated and have made substantial contributions to the approval of the final version. DL contributed to the conception and design of the work. KH, CV, HA-Ö, and JK contributed to the acquisition and analysis of data. DL and KH contributed to the interpretation of data for the work. DL drafted the work and all authors revised it critically for important intellectual content.

## Conflict of Interest Statement

The authors declare that the research was conducted in the absence of any commercial or financial relationships that could be construed as a potential conflict of interest.

## Footnotes

1 www.ECAS.network

## References

[B1] Abrahams S. Newton J. Niven E. Foley J. Bak T. H. (2014). Screening for cognition and behaviour changes in ALS. *Amyotroph. Lateral Scler. Frontotemporal Degener.* 15 9–14. 10.3109/21678421.2013.805784 23781974

[B2] Beeldman E. Raaphorst J. Twennaar M. K. de Visser M. Schmand B. A. de Haan R. J. (2016). The cognitive profile of ALS: a systematic review and meta-analysis up-date. *J. Neurol. Neurosurg. Psychiatry* 87 611–619. 10.1136/jnnp-2015-310734 26283685

[B3] Cedarbaum J. M. Stambler N. Malta E. Fuller C. Hilt D. Thurmond B. (1999). The ALSFRS-R: a revised ALS functional rating scale that in-corporates assessments of respiratory function. *J. Neurol. Sci.* 169 13–21. 10.1016/S0022-510X(99)00210-5 10540002

[B4] Chaudhary U. Birbaumer N. Ramos-Murguialday A. (2016). Brain-computer interfaces for communication and rehabilitation. *Nat. Rev. Neurol.* 12 513–525. 10.1038/nrneurol.2016.113 27539560

[B5] Choularton S. Dale R. (2004). “User responses to speech recognition errors: consistency of behaviour across domains,” in *Proceedings of the 10th Australian International Conference on Speech Science and Technology* Sydney, NSW.

[B6] Cipresso P. Carelli L. Solca F. Meazzi D. Meriggi P. Poletti B. (2012). The use of P300-based BCIs in amyotrophic lateral sclerosis: from augmentative and alternative communication to cognitive assessment. *Brain Behav.* 2 479–498. 10.1002/brb3.57 22950051 PMC3432970

[B7] Duvinage M. Castermans T. Petieau M. Hoellinger T. Cheron G. Dutoit T. (2013). Performance of the Emotiv Epoc headset for P300-based applications. *Biomed. Eng. Online* 12:56. 10.1186/1475-925X-12-56 23800158 PMC3710229

[B8] Elamin M. Bede P. Byrne S. Jordan N. Gallagher L. Wynne B. (2013). Cognitive changes predict functional decline in ALS: a population-based longitudinal study. *Neurology* 80 1590–1597. 10.1212/WNL.0b013e31828f18ac 23553481

[B9] Farwell L. A. Donchin E. (1988). Talking off the top of your head: toward a mental pros-thesis utilizing event-related brain potentials. *Electroencephalogr. Clin. Neurophysiol.* 70 510–523. 10.1016/0013-4694(88)90149-6 2461285

[B10] Fuchino Y. Nagao M. Katura T. Bando M. Naito M. Maki A. (2008). High cognitive function of an ALS patient in the totally locked-in state. *Neurosci. Lett.* 435 85–89. 10.1016/j.neulet.2008.01.046 18359565

[B11] Goldstein L. H. Abrahams S. (2013). Changes in cognition and behaviour in amyotrophic lateral sclerosis: nature of impairment and implications for assessment. *Lancet Neurol.* 12 368–380. 10.1016/S1474-4422(13)70026-7 23518330

[B12] Hammer E. M. Häcker S. Hautzinger M. Meyer T. D. Kübler A. (2008). Validity of the ALS-Depression-Inventory (ADI-12)–a new screening instrument for depressive disorders in patients with amyotrophic lateral sclerosis. *J. Affect. Disord.* 109 213–219. 10.1016/j.jad.2007.11.012 18262283

[B13] Kasper E. Zydatiss K. Schuster C. Machts J. Bittner D. Kaufmann J. (2016). No change in executive performance in ALS patients: a longitudinal neuro-psychological study. *Neurodegen. Dis.* 16 184–191. 10.1159/000440957 26613522

[B14] Kaufmann T. Holz E. M. Kübler A. (2013). Comparison of tactile, auditory, and visual modality for brain-computer interface use: a case study with a patient in the locked-in state. *Front. Neurosci.* 7:129. 10.3389/fnins.2013.00129 23898236 PMC3721006

[B15] Keller J. Gorges M. Horn H. T. Aho-Özhan H. E. Pinkhardt E. H. Uttner I. (2015). Eye-tracking controlled cognitive function tests in patients with amyotrophic lateral sclerosis: a controlled proof-of-principle study. *J. Neurol.* 262 1918–1926. 10.1007/s00415-015-7795-3 26041615

[B16] Keller J. Krimly A. Bauer L. Schulenburg S. Böhm S. Aho-Özhan H. E. A. (2017). A first approach to a neuropsychological screening tool using eye-tracking for bedside cognitive testing based on the Edinburgh Cognitive and Behavioural ALS Screen. *Amyotroph. Lateral Scler. Frontotemporal Degener.* 18 443–450. 10.1080/21678421.2017.1313869 28420245

[B17] Kotchoubey B. Lang S. Winter S. Birbaumer N. (2003). Cognitive processing in completely paralyzed patients with amyotrophic lateral sclerosis. *Eur. J. Neurol.* 10 551–558. 10.1046/j.1468-1331.2003.00647.x 12940838

[B18] Kübler A. Winter S. Kaiser J. Birbaumer N. Hautzinger M. (2005). Das ALS-Depressionsinventar (ADI): Ein Fragebogen zur Messung von Depression bei dege-nerativen neurologischen Erkrankungen (amyotrophe Lateralsklerose). *Zeitschrift für Klinische Psychologie und Psychotherapie* 34 19–26. 10.1026/1616-3443.34.1.19

[B19] Lakerveld J. Kotchoubey B. Kübler A. (2008). Cognitive function in patients with late stage amyotrophic lateral sclerosis. *J. Neurol. Neurosurg. Psychiatry* 79 25–29. 10.1136/jnnp.2007.116178 17519321

[B20] Loose M. Burkhardt C. Aho-Özhan H. Keller J. Abdulla S. Böhm S. (2016). Age and education-matched cut-off scores for the revised German/Swiss-German version of ECAS. *Amyotroph. Lateral Scler. Frontotemporal Degener.* 17 374–376. 10.3109/21678421.2016.1162814 27027323

[B21] Lulé D. Burkhardt C. Abdulla S. Böhm S. Kollewe K. Uttner I. (2015). The Edinburgh cognitive and behavioural amyotrophic lateral sclerosis screen: a cross-sectional comparison of established screening tools in a German-Swiss population. *Amyotroph. Lateral Scler. Frontotemporal Degener.* 16 16–23. 10.3109/21678421.2014.959451 25292386

[B22] Marchetti M. Priftis K. (2014). Effectiveness of the P3-speller in brain–computer interfaces for amyotrophic lateral sclerosis patients: a systematic review and meta-analysis. *Front. Neuroeng.* 7:12. 10.3389/fneng.2014.00012 24847247 PMC4013458

[B23] McCane L. M. Heckman S. M. McFarland D. J. Townsend G. Mak J. N. Sellers E. W. (2015). P300-based brain-computer interface (BCI) event-related potentials (ERPs): people with amyotrophic lateral sclerosis (ALS) vs. *age-matched controls*. *Clin. Neurophysiol.* 126 2124–2131. 10.1016/j.clinph.2015.01.013 25703940 PMC4529383

[B24] Mugler E. M. Ruf C. A. Halder S. Bensch M. Kubler A. (2010). Design and implementation of a P300-based brain–computer interface for controlling an internet browser. *IEEE Trans. Neural Syst. Rehabil. Eng.* 18 599–609. 10.1109/TNSRE.2010.2068059 20805058

[B25] Münßinger J. I. Halder S. Kleih S. C. Furdea A. Raco V. Hösle A. (2010). Brain painting: first evaluation of a new brain–computer interface application with ALS-patients and healthy volunteers. *Front. Neurosci.* 4:182 10.3389/fnins.2010.00182 PMC2996245 21151375

[B26] Nijboer F. Sellers E. W. Mellinger J. Jordan M. A. Matuz T. Furdea A. (2008). A P300-based brain–computer interface for people with amyotrophic lateral sclerosis. *Clin. Neurophysiol.* 119 1909–1916. 10.1016/j.clinph.2008.03.034 18571984 PMC2853977

[B27] Niven E. Newton J. Foley J. Colville S. Swingler R. Chandran S. (2015). Validation of the Edinburgh Cognitive and Behavioural Amyotrophic Lateral Sclerosis Screen (ECAS): a cognitive tool for motor disorders. *Amyotroph. Lateral Scler. Frontotemporal Degener.* 16 172–179. 10.3109/21678421.2015.1030430 25967542

[B28] Ogawa T. Tanaka H. Hirata K. (2009). Cognitive deficits in amyotrophic lateral sclerosis evaluated by event-related potentials. *Clin. Neurophysiol.* 120 659–664. 10.1016/j.clinph.2009.01.013 19342291

[B29] Phukan J. Elamin M. Bede P. Jordan N. Gallagher L. Byrne S. (2012). The syndrome of cognitive impairment in amyotrophic lateral sclerosis: a population-based study. *J. Neurol. Neurosurg. Psychiatry* 83 102–108. 10.1136/jnnp-2011-300188 21836033

[B30] Poletti B. Carelli L. Solca F. Lafronza A. Pedroli E. Faini A. (2016). Cognitive assessment in amyotrophic lateral sclerosis by means of P300-brain com-puter interface: a preliminary study. *Amyotroph. Lateral Scler. Frontotemporal Degener.* 17 473–481. 10.1080/21678421.2016.1181182 27169693

[B31] Schalk G. McFarland D. J. Hinterberger T. Birbaumer N. Wolpaw J. R. (2004). BCI2000: a general-purpose brain-computer interface (BCI) system. *IEEE Trans. Biomed. Eng.* 51 1034–1043. 10.1109/TBME.2004.827072 15188875

[B32] Schmidt-Atzert L. Amelang M. (2012). *Psychologische Diagnostik*, 5th Edn. Berlin: Springer 10.1007/978-3-642-17001-0

[B33] Schreiber H. Gaigalat T. Wiedemuth-Catrinescu U. Graf M. Uttner I. Muche R. (2005). Cognitive function in bulbar- and spinal- onset amyotrophic lat-eral sclerosis: a longitudinal study in 52 patients. *J. Neurol.* 252 772–781. 10.1007/s00415-005-0739-6 15742104

[B34] Trojsi F. Santangelo G. Caiazzo G. Siciliano M. Ferrantino T. Piccirillo G. (2016). Neuropsychological assessment in different King’s clinical stages of amyotrophic lateral sclerosis. *Amyotroph. Lateral Scler. Frontotemporal Degener.* 17 228–235. 10.3109/21678421.2016.1143513 26905940

[B35] Wei Q. Chen X. Zheng Z. Huang R. Guo X. Cao B. (2015). Screening for cognitive impairment in a Chinese ALS population. *Amyotroph. Lateral Scler. Frontotemporal Degener.* 16 40–45. 10.3109/21678421.2014.966311 25309978

[B36] Xu Z. Alruwaili A. R. S. Henderson R. D. McCombe P. A. (2017). Screening for cognitive and behavioural impairment in amyotrophic lateral sclerosis: frequency of ab-normality and effect on survival. *J. Neurol. Sci.* 376 16–23. 10.1016/j.jns.2017.02.061 28431606

